# Impact of Renal Function on Effectiveness and Safety Associated With Low Dose Dabigatran in Non-valve Atrial Fibrillation Patients After Catheter Ablation

**DOI:** 10.3389/fcvm.2021.762872

**Published:** 2021-10-28

**Authors:** Xiaoye Li, Chengchun Zuo, Qiuyi Ji, Zi Wang, Qianzhou Lv

**Affiliations:** Department of Pharmacy, Zhongshan Hospital, Fudan University, Shanghai, China

**Keywords:** effectiveness and safety outcomes, evaluated glomerular filtration rate, non-valve atrial fibrillation, dabigatran, composite endpoint

## Abstract

**Aim:** The purpose of this study is to compare the effectiveness and safety of 110 mg dabigatran in non-valve atrial fibrillation (NVAF) patients with different eGFRs.

**Methods:** We conducted a single-center retrospective cohort study to investigate the effectiveness and safety of 110 mg dabigatran for NVAF patients between January 2017 and December 2018 based on the eGFR category.

**Results:** A total of 560 NVAF patients who treated with 110 mg dabigatran were included for analysis. In 12 months, the Kaplan-Meier survival curves indicated that the lower eGFR subgroups were more likely to experience thrombosis, bleeding, and cumulative events earlier (*P* = 0.021 for thrombosis; *P* = 0.026 for bleeding; *P* = 0.001 for cumulative events). Gastrointestinal bleeding occurred more frequently in the moderate group than in other groups (6.94% in the moderate group vs. 1.54% in the mild group vs. 1.22% in the normal group, *P* = 0.028). By multivariate analysis, chronic kidney disease (*P* = 0.043; OR = 4.273, 95% CI 1.043–17.543) and diabetes mellitus (*P* = 0.023; OR = 2.194, 95% CI 1.114–4.323) were independent predictors of the composite endpoints. A positive linear relationship was observed between eGFR levels and occurrence rate of thrombosis and bleeding under anticoagulation patients with 110 mg dabigatran (*R*^2^ = 0.432 and *R*^2^ = 0.784, respectively).

**Conclusions:** Impaired renal function was associated with decreased safety and increased thrombosis risks in NVAF patients taking low dose dabigatran.

## Introduction

Non-valve atrial fibrillation (NVAF) is one class of supra ventricular tachyarrhythmia with uncoordinated atrial electrical activation and consequently causes ineffective atrial contraction which can result in hemodynamic abnormalities and thromboembolic events contributing to severe morbidity and mortality (1). Currently, the guidelines recommend the use of dabigatran for the prevention of thrombosis in NVAF patients, and it is usually not necessary to monitor the coagulation function during the medication.

Chronic kidney disease (CKD) is defined as the presence of kidney damage or decreased level of kidney function [(evaluated glomerular filtration rate, eGFR) <60 mL/min/1.73m^2^] for 3 months or more, and may have a higher risk of AF than the general population (2, 3). In addition, patients who need direct oral anticoagulation (DOAC) for thrombosis prevention are considered to be at high risk of adverse events related to renal dysfunction (4). Meanwhile, abnormal renal function already had been considered a risk factor for the fully validated HAS-BLED score (1).

Dabigatran has now been widely used to prevent stroke and systemic embolism in NVAF patients. The clinical trial (5) indicated that age, weight, renal function and concomitant antiplatelet therapy had an effect on anticoagulation. Since large proportion of dabigatran (~80%) is excreted by the kidneys (6), renal dysfunction may lead to drug accumulation, which increases the risk of bleeding events. It should be mentioned that previous studies (7, 8) focused on the comparison of efficacy and safety of warfarin and dabigatran in NVAF patients with different renal functions. Only a few studies (5) compared the clinical outcomes of dabigatran itself with different eGFRs.

In recent years, dabigatran had altered the anticoagulation pattern of stroke prevention in people with atrial fibrillation. Clinical pharmacokinetic studies indicated that dabigatran plasma concentration might increase under standard fixed dosing in CKD patients with the decrease of renal clearance, thus may lead to different levels of safety and effectiveness. At the same time, there is a lack of data on the clinical benefits and risks of low dose dabigatran among AF patients with different renal functions. The purpose of this study is to compare the effectiveness and safety of low dose dabigatran on NVAF patients with different eGFRs.

## Methods

### Study Design and Patient Population

We conducted a single-center retrospective cohort study to investigate the effectiveness and safety of low dose dabigatran (110 mg, available dose) among the enrolled patients diagnosed with NVAF in the department of cardiology, Zhongshan Hospital, Fudan University between January 2017 and December 2018. Medical Ethics Committee of Zhongshan Hospital approved this study. The requirement for informed consent was waived by the ethics committee because this retrospective analysis was limited to preexisting data from medical records, collected as parts of the standard-treatment by physicians. Indentified data were anonymized and privacy issues were kept confidential.

The NVAF was diagnosed according to the European Society of Cardiology (ESC) criteria (1) and all the patients newly underwent an electrocardiogram (ECG) exam showing a typical AF pattern: absolutely irregular RR intervals with no discernible, distinct P waves. All patients underwent catheter ablation of AF successfully, and were subsequently remained in sinus rhythm. The renal functions were defined as the classification of estimated glomerular filtration rate (eGFR) calculated by the CKD-EPI formula. According to the KDOQI guidelines (9), participants were stratified into three groups: (1) the normal renal function group defined as eGFR beyond 90 mL/min/1.73m^2^; (2) the mildly decreased renal function group defined as eGFR ranging from 60 to 89 mL/min/1.73m^2^; (3) the moderately impaired renal function group defined as eGFR between 30 and 59 mL/min/1.73m^2^.

To be enrolled in this study, patients must be at risk of stroke, transient ischemic attack, and systemic embolism with CHA_2_DS_2_-VASc score ≥1, and received dabigatran (110 mg bid, the only available dosage) as part of stroke/systemic embolism risk reduction management. The main exclusion criteria included the following: (1) history of bleeding and hemorrhagic diseases; (2) discontinuation of dabigatran anticoagulation; (3) lack of follow-ups; (4) severely impaired renal impairment function and kidney failure (eGFR ≤ 30 mL/min·1.73 m^2^) or patients in need of dialysis.

### Laboratory Measurements

Detailed information on demographic, diagnosis, history of smoking or alcohol consumption, comorbidities, levels of hemoglobin (Hb) and platelet (PLT) count, alanine aminotransferase and indicators of coagulation function as well as concomitant drugs in use about each patient was collected through electronic medical records. Predetermined stroke and bleeding risk was assessed by the CHA_2_DS_2_-VAS_C_ and HAS-BLED score (10).

### Follow-Ups

Routine follow-ups including a 12-lead ECG and 24-holter monitoring were performed by cardiologists or specialists at each outpatient visit. ECG and 24-holter monitoring in AF patients were mainly used to evaluate the adequacy of heart rate control and the symptoms associated with AF recurrence, and to detect the focal induction of paroxysmal AF. Additional transthoracic echocardiography was carried out to identify thrombosis formation in the left atrium and to assess the size and function of the left ventricular (systolic and diastolic), atrial size, and right heart function during post-anticoagulation. The study endpoints were pooled via data and reports from the medical records system, including clinical characteristics, laboratory data, occurrence of thrombotic and bleeding events during 12-month follow-ups.

### Clinical Outcomes

The endpoints were adjudicated by an independent endpoint committee blinded for the renal function of the patients. The primary effectiveness clinical outcomes were defined as the recurrence of systemic embolism, including stroke diagnosed neurologically by computed tomography (CT) or magnetic resonance imaging (MRI), pulmonary embolism, venous thromboembolism, and cardiac embolism during the 12-month follow-up period. The principal safety goals were to compare the incidence of major and clinically relevant non-major bleeding complications concordant with the guidance by the International Society on Thrombosis and Haemostasis (10) such as gastrointestinal hemorrhage, hematemesis, epistaxis, operation site hemorrhage, and bleeding gums. Event-free complications were defined as the time from enrollment to occurrence of systemic embolism and bleeding complications, whichever occurred first. The events were screened for a period of 12 months from enrollment to completion of data collection.

Routine blood tests, coagulation function tests and concomitant medications were recorded. A blood coagulation test was conducted at least 48 h after the first re-dose of dabigatran. The potential bleeding level was defined as prothrombin time (PT) longer than 13 s, or activated partial thromboplastin time (APTT) longer than 31.3 s. Various indicators of cardiac structure and function under cardiac ultrasound were evaluated during the follow-ups.

### Statistical Analysis

The descriptive statistics of continuous variables were expressed as means ± standard deviations (SD), while those of discrete variables were expressed as counts or percentages. Two-way ANOVA was applied to compare the differences of the continuous variables among the four groups of patients, and a chi-squared test was conducted to compare the distribution of categorical variables.

The comparison of thrombosis and bleeding complications were analyzed by carrying out chi-squared test. We adopted Kaplan-Meier method to survival curve analysis by using log-rank test for trend and the Cox regression analysis. We compared the time to thrombosis (defined as the time from enrollment to the first occurrence of a stroke or systemic embolism, TTT), time to bleeding (defined as the time from enrollment to the first occurrence of bleeding, TTB), and event-free outcome (defined as the time from enrollment to the first occurrence of thrombosis or bleeding) among the three groups.

We analyzed the association between the composite endpoint and eGFR categories by using multiple statistical models. Variables including potential thrombosis and bleeding risk factors were analyzed. Odds ratios (ORs) with two-sided 95% confidence intervals (CIs) were calculated for the risk factors of the composite endpoint. The results were presented as ORs along with 95% CI. Two-sided *P*-values were used to determine significance (threshold, *P* < 0.05). Statistical analysis was conducted using SPSS (IBM SPSS Statistics 22.0) and Prism 5 (GrandPad Software). A *P*-value of 0.05 was considered as the threshold for statistical significance.

## Results

### Patients' Characteristics

**Figure 1:**
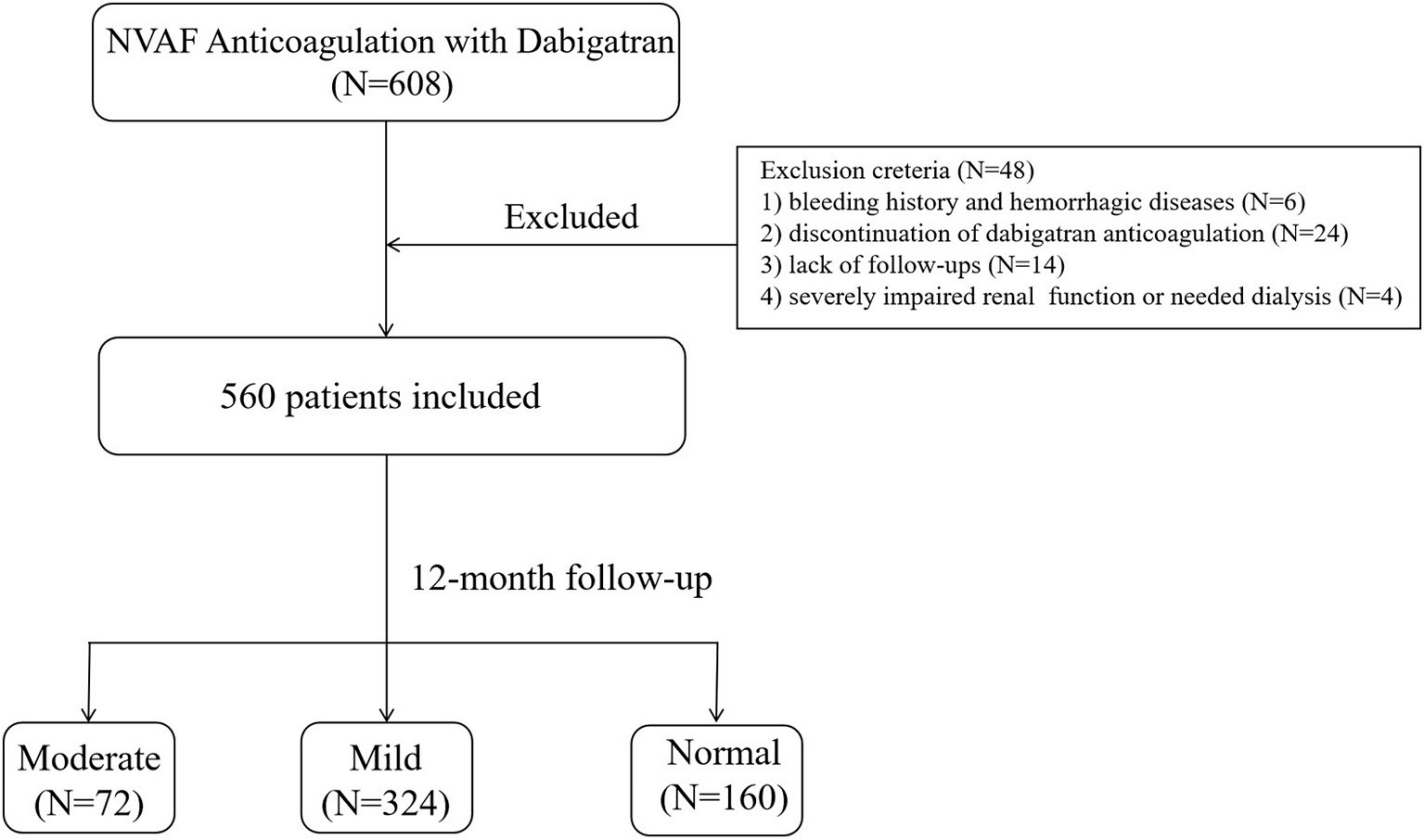
Diagrammatic presentation of our study.

During the study period, a total of 608 NVAF patients who underwent catheter ablation and treated with dabigatran were included in our study. After applying the exclusion criteria, 48 (7.9%) patients were omitted from the study. Of those excluded, 6 were with bleeding history and hemorrhagic disease, 14 lacked follow-ups, 24 discontinued with dabigatran, and 4 were with severe impaired renal function or needed dialysis (Figure 1). A total of 560 patients were included for analysis. According to the eGFR category, 72 (12.8%) patients had moderate renal impairment (eGFR, 30–59 mL/min·1.73 m^2^), 324 (57.8%) had mild renal impairment (eGFR, 60–89 mL/min·1.73 m^2^), and 164 (29.4%) had normal renal function (eGFR, ≥90 mL/min·1.73m^2^).

Table 1 summarized the basic demographic and clinical characteristics of the patients. There were no significant differences in the three renal function subgroups regarding sex, age, comorbidities, laboratory indicators, concomitant medications, predetermined stroke risk [CHA_2_DS_2_-VAS_C_ (P=0.430)] or predetermined major bleeding risk [HAS-BLED (*P* = 0.590)].

**Table 1:** Baseline characteristics stratified according to the eGFR category.

**Baseline characteristics**	**Total**	**Moderate**	**Mild**	**Normal**	***P*-value**
	**(*n* = 560)**	**(*n* = 72)**	**(*n* = 324)**	**(n = 164)**	
Age, mean, y (SD)	67.9 (8.2)	68.7 (7.9)	68.2 (9.0)	67.1 (6.7)	0.455
BMI, kg/m^2^; mean (SD)	24.6 (3.3)	24.9 (3.5)	24.6 (3.2)	24.6 (3.4)	0.895
Gender (% male)	352 (62.9%)	44 (61.1%)	205 (63.3%)	103 (62.8%)	0.990
Smoking, *n* (%)	100 (17.9%)	9 (12.5%)	54 (16.7%)	37 (22.6%)	0.241
Alcohol, *n* (%)	64 (11.4%)	4 (5.6%)	36 (11.1%)	24 (14.6%)	0.246
**Co-morbidity**					
Hypertension	304 (54.3%)	41 (56.9%)	183 (56.5%)	80 (48.8%)	0.417
Dyslipidemia, *n* (%)	178 (31.8%)	21 (29.2%)	104 (32.1%)	53 (32.3%)	0.967
Diabetes, *n* (%)	152 (27.1%)	27 (37.5%)	86 (26.5%)	39 (23.8%)	0.179
Stroke/TIA, *n* (%)	88 (15.7%)	14 (19.4%)	57 (17.6%)	17 (10.4%)	0.160
Liver disease, *n* (%)	50 (8.9%)	5 (6.9%)	30 (9.3%)	15 (9.1%)	0.940
Cancer, *n* (%)	10 (1.8%)	1 (1.4%)	6 (1.8%)	3 (1.8%)	0.961
Heart failure, *n* (%)	107 (19.1%)	16 (22.2%)	69 (21.3%)	22 (13.4%)	0.180
PAD, *n* (%)	18 (3.2%)	4 (5.6%)	11 (3.4%)	3 (1.8%)	0.510
AVB, *n* (%)	51 (9.1%)	8 (11.1%)	26 (8.0%)	17 (10.4%)	0.772
**Laboratory test**					
LDL, mmol/L; mean (SD)	2.3 (0.84)	2.1 (0.89)	2.3 (0.83)	2.4 (0.81)	0.115
Hb, g/L; mean (SD)	136.1 (16.6)	133.2 (18.7)	136.0 (16.4)	137.6 (16.6)	0.952
PLT, ^*^10^9^/L; mean (SD)	192.1 (56.5)	189.6 (62.6)	188.3 (55.0)	200.7 (56.2)	0.143
ALT, U/L; mean (SD)	25.4 (24.1)	24.8 (19.6)	24.2 (23.1)	27.7 (27.7)	0.519
Hemoglobin A1c, %; mean (SD)	6.0 (1.0)	6.2 (0.9)	6.1 (1.1)	6.0 (0.9)	0.583
APTT, s; mean (SD)	30.3 (5.5)	30.5 (3.8)	30.4 (6.0)	30.0 (5.1)	0.756
PT, s; mean (SD)	12.6 (2.5)	12.6 (2.2)	12.8 (2.4)	12.3 (2.7)	0.276
TT, s; mean (SD)	32.5 (34.8)	32.2 (33.4)	33.0 (37.8)	31.5 (28.6)	0.978
D-Dimer, mg/L; mean (SD)	0.47 (1.2)	0.48 (1.0)	0.46 (1.4)	0.48 (0.8)	0.999
**Concomitant medication**					
Statin, *n* (%)	190 (33.9%)	22 (30.6%)	114 (35.2%)	54 (32.9%)	0.881
Anti-hypertension agent, *n* (%)	326 (58.2%)	44 (61.1%)	191 (59.0%)	91 (55.5%)	0.844
β-blockers, *n* (%)	115 (20.5%)	18 (25.0%)	63 (19.4%)	34 (20.7%)	0.772
Antiplatelet, *n* (%)	155 (27.7%)	27 (37.5%)	88 (27.2%)	40 (24.4%)	0.221
Anti-arrhythmic agent, *n* (%)	49 (8.8%)	9 (12.5%)	23 (7.1%)	17 (10.4%)	0.406
PPI, *n* (%)	62 (11.1%)	7 (9.7%)	34 (10.5%)	21 (12.8%)	0.863
**Thrombosis and bleeding potential risk score**					
^*^CHA_2_DS_2_-VASc; mean (SD)	2.8 (1.6)	2.9 (1.3)	3.0 (1.7)	2.7 (1.4)	0.430
CHA_2_DS_2_-VASc ≥ 2, *n* (%)	422 (75.4%)	61 (84.4%)	234 (72.2%)	127 (77.4%)	0.139
CHA_2_DS_2_-VASc ≥ 3, *n* (%)	292 (52.1%)	43 (59.7%)	173 (53.4%)	76 (46.3%)	0.254
CHA_2_DS_2_-VASc ≥ 4, *n* (%)	174 (31.1%)	23 (31.9%)	110 (34.0%)	41 (25.0%)	0.251
^#^HAS-BLED; mean (SD)	2.0 (1.0)	2.2 (1.1)	2.0 (1.0)	2.0 (0.9)	0.590
HAS-BLED ≥ 3, *n* (%)	219 (39.1%)	32 (44.4%)	126 (38.9%)	61 (37.2%)	0.772

*The data is displayed as mean (SD) or %. SD, standard deviation. P-value was calculated with two-way ANOVA analysis; Moderate represented with eGFR between 30 and 59 mL/min·1.73 m^2^; Mild represented with eGFR between 60 and 89 mL/min 1.73m^2^; Normal represented with eGFR ≥ 90 mL/min·1.73 m^2^; BMI, body mass index; TIA, transient ischemic attack; PAD, peripheral artery disease; AVB, Atrioventricular block; LDL, low-density lipoprotein; Hct, hematocrit; Hb, hemoglobin; PLT, platelet; ALT, alanine aminotransferase; CK-MB, creatine kinase-MB; NT-proBNP, N-terminal pronatriuretic peptide; APTT, activated partial thromboplastin time; PT, prothrombin time; TT, Thrombin time; INR, international normalized ratio; PPI, proton pump inhibitor*.

* *CHA2DS2-VASc score (total 0–9 points) was determined by: 1 point for each of congestive heart failure/left ventricular dysfunction, hypertension, diabetes mellitus, vascular disease, female gender, age 65–74 years; 2 points for each of age ≥75 years, previous stroke/transient ischemic attack/thromboembolism*.

# *HAS-BLED score (total 0–9 points) was determined by: 1 point for each of hypertension, renal dysfunction, hepatic dysfunction, previous stroke, bleeding history/predisposition, labile INR, age > 65 years, concomitant use of antiplatelet drugs, alcohol excess*.

### Assessment of Clinical Outcomes Among Different Renal Function Categories During Medication With Dabigatran

During the 12-month follow-up, there were significant differences in the incidence of systemic thromboembolism among the three groups, which was higher in the moderately impaired renal group (moderate 8.3% vs. mild 3.4% vs. normal 1.2%; ^*^P = 0.021; Table 2). No significant difference was found in the incidence of left atrial dilation, which was defined as enlargement of left atrial diameter (LAD >40 mm) and heart failure (LVEF <40%), among the three groups (*P* > 0.05).

**Table 2:** Assessment of clinical outcomes among different renal function categories.

	**Moderate**	**Mild**	**Normal**	***P*-value**
	**(*n* = 72)**	**(*n* = 324)**	**(n = 164)**	
Systemic thrombosis, *n* (%)	6 (8.3%)	11 (3.4%)	2 (1.2%)	0.021^*^
VTE, *n* (%)	3 (4.2%)	3 (0.9%)	0 (0.0%)	0.150
PE, *n* (%)	2 (2.8%)	4 (1.2%)	0 (0.0%)	0.147
Stroke, *n* (%)	0 (0.0%)	0 (0.0%)	1 (0.6%)	0.298
Cardiac thrombosis, *n* (%)	1 (1.4%)	4 (1.2%)	1 (0.6%)	0.787
LAD > 40 mm, *n* (%)	65 (90.3%)	294 (90.7%)	147 (89.6%)	0.926
LVEF <40%, *n* (%)	5 (6.9%)	17 (5.2%)	8 (4.9%)	0.794

*VTE, venous thromboembolism; PE, pulmonary embolism; LAD, left atrial dimension; LVEF, left ventricular ejection fraction*.

* *P < 0.05*.

*Systemic thromboembolism is defined as pulmonary embolism, venous thromboembolism, or cardiac embolism*.

**Figure 2:**
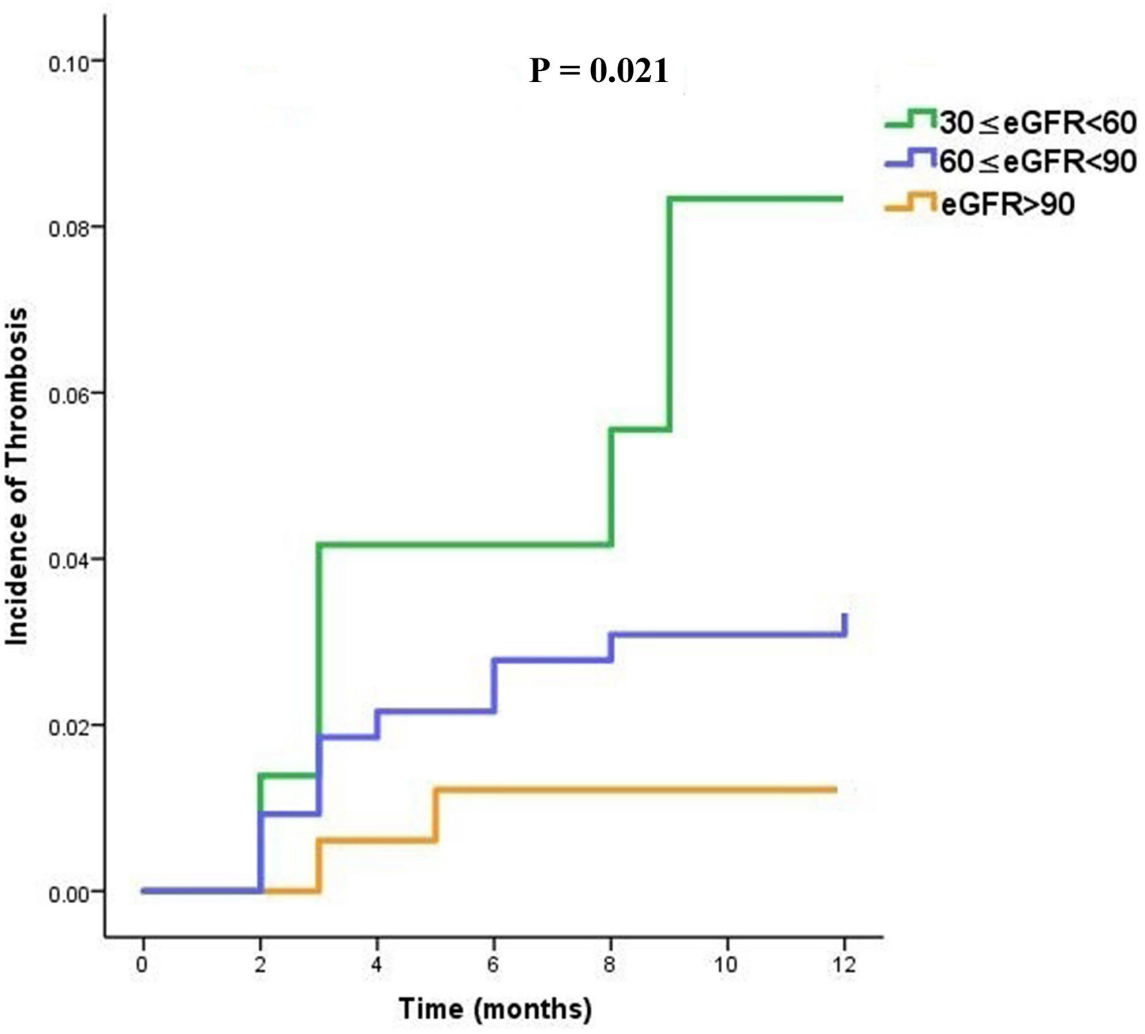
Time to thrombosis (TTT) curves in dabigatran treated patients, stratified into three subgroups (moderate, mild, and normal) according to the eGFR category.

During the 12-month follow-up period, a total of 19 (3.4%) patients experienced systemic thrombotic events. The Cumulative Kaplan-Meier estimates illustrated that the incidence of the primary effectiveness endpoint of systemic thrombosis was lower in normal renal function patients and the difference was statistically significant compared with patients with moderate and mild renal impairment (*P* = 0.021; Figure 2). In the whole cohort, those with moderate renal impairment were more likely to have a shorter time to thrombosis (TTT) as demonstrated in Figure 2.

### Anticoagulation-Related Complications

The total frequencies of bleeding events were shown in Table 3. Overall, the most common bleeding event was gastrointestinal bleeding, which occurred more frequently in the moderate group than those in the mild and normal groups (6.94% moderate vs. 1.54% mild vs. 1.22% normal), and a significant difference was found among the three groups (*P* = 0.028).

**Table 3:** Bleeding complications comparison within 12-month follow-ups.

**Bleeding events**	**Moderate**	**Mild**	**Normal**	***P*-value**
	**(*n* = 72)**	**(*n* = 324)**	**(n = 164)**	
Gastrointestinal hemorrhage, *n* (%)	5 (6.94%)	5 (1.54%)	2 (1.22%)	0.028^*^
Hematuria, *n* (%)	0 (0.0%)	3 (0.93%)	1 (0.61%)	1.000
Operation site hemorrhage, *n* (%)	1 (1.39%)	4 (1.23%)	0 (0.0%)	0.326
Bleeding gums, *n* (%)	0.00	2 (0.62%)	0 (0.0%)	0.658
Skin ecchymosis, *n* (%)	2 (2.78%)	5 (1.54%)	0 (0.0%)	0.112
PLT <125 s, *n* (%)	9 (12.50%)	36 (11.11%)	13 (7.93%)	0.450
Male: Hb <120 g/L Female: Hb <110 g/L, *n* (%)	9 (12.50%)	24 (7.41%)	12 (7.32%)	0.328
APTT > 31s, *n* (%)	25 (34.72%)	120 (37.04%)	59 (35.98%)	0.925
PT > 13s, *n* (%)	9 (12.50%)	41 (12.65%)	14 (8.54%)	0.383

*PLT, platelet; Hb, hemoglobin; PT, prothrombin time*.

* *P < 0.05*.

Moreover, operation site hemorrhage occurred less frequently in the normal group than that in the moderate and mild groups, but no significant difference was found amidst groups (*P* = 0.326). The cumulative incidence of bleeding complications such as hematuria, skin ecchymosis and bleeding gums during anticoagulation therapy was similar in the three groups (*P* > 0.05). There was no significant difference among the three groups with respect to the levels of Hb, PLT, and PT under the coagulation threshold (*P* > 0.05).

**Figure 3:**
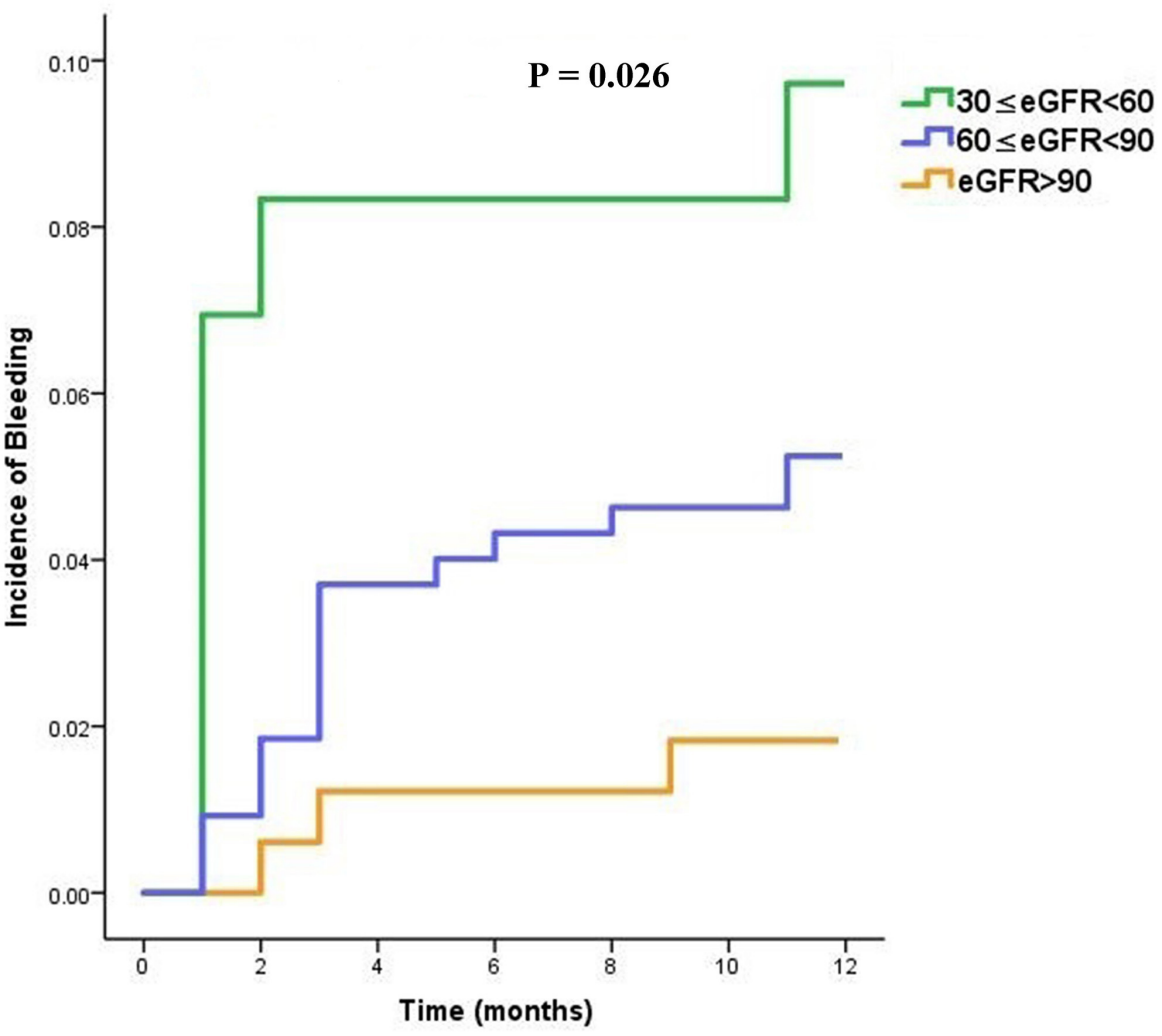
Time to bleeding (TTB) curves in dabigatran treated patients, stratified into three subgroups (moderate, mild, and normal) according to the eGFR category.

Similarly, considering whole cohort of patients, those with moderate renal impairment were more likely to experience bleeding sooner when treated with dabigatran (overall *P* = 0.026) as shown in Figure 3.

### Analysis of Composite Endpoint in Patients With Impaired Renal Function in Comparison to Patients With Normal Renal Function

A total of 45 (8.0%) patients receiving dabigatran experienced either thrombosis or bleeding, and we observed no substantial difference in the composite endpoint among the three groups (overall *P* = 0.020, with 12-month composite endpoint rates of 16.7, 8.6, and 3.0% for moderately impaired, mildly impaired, and normal renal function patients, respectively).

**Figure 4:**
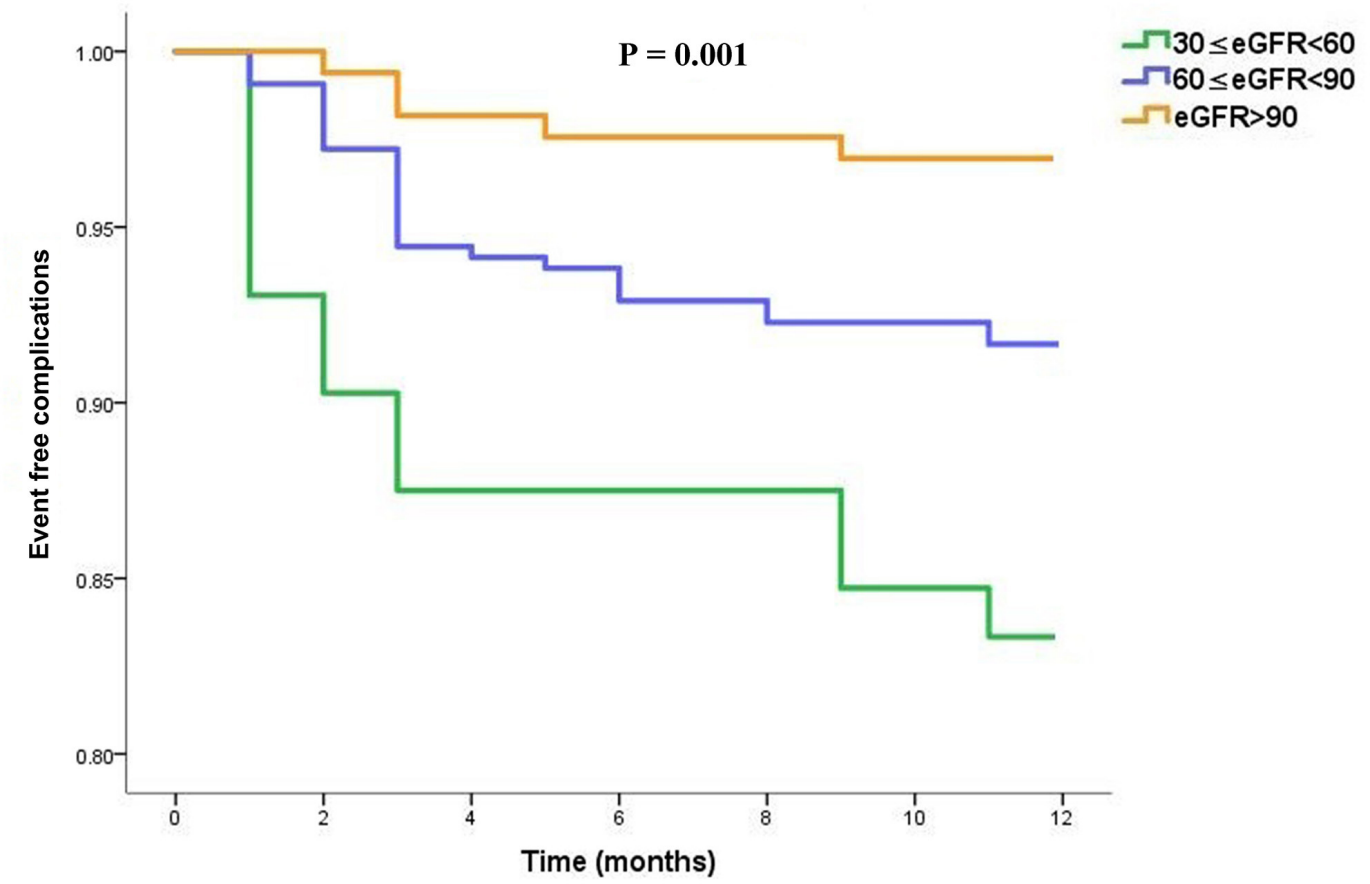
Event-free complications curves in dabigatran treated patients, stratified into three subgroups (moderate, mild and normal) according to the eGFR category.

We further performed the same analysis to compare event-free outcomes among the three subgroups. Regarding the event-free outcomes for dabigatran, the cumulative events differed according to the eGFR category. Patients with lower eGFR were more likely to experience a cumulative event earlier (overall *P* = 0.001) as shown in Figure 4.

Multivariate logistic regression was performed to identify the independent association between the composite endpoints and the potential risk factors of CHA_2_DS_2_-VASc and HAS-BLED scores. According to multivariate analysis, chronic kidney disease (CKD) (*P* = 0.043; OR = 4.273, 95% CI 1.043–17.543) and diabetes mellitus (DM) (*P* = 0.023; OR = 2.194, 95% CI 1.114–4.323) were identified as independent predictors of the composite endpoints of dabigatran treatment, as shown in Table 4.

**Table 4:** Association of the CHA_2_DS_2_-VASc and HAS-BLED score potential risk factors with composite endpoint in dabigatran-treated patients.

**Variables**	**SE**	***P*-value**	**OR**	**95% CI**
Gender (male)	0.353	0.899	0.956	0.479–1.910
Age > 65 (y)	0.363	0.738	0.885	0.434–1.805
CKD	0.720	0.043^*^	4.273	1.043–17.543
Smoke	0.528	0.836	0.896	0.318–2.524
Alcohol	0.713	0.604	0.691	0.171–2.795
HTN	0.345	0.693	1.146	0.583–2.255
DM	0.346	0.023^*^	2.194	1.114–4.323
Liver dysfunction	1.036	0.166	0.238	0.031–1.815
HF	0.410	0.863	1.073	0.480–2.397
Stroke	0.419	0.381	1.444	0.635–3.287
PAD	1.080	0.565	0.537	0.065–4.457
Antiplatelet	0.367	0.695	0.866	0.422–1.776

*CKD, chronic kidney disease (eGFR <60 mL/min 1.73 m^2^); HTN, Hypertension; DM, diabetes mellitus; HF, Heart Failure; PAD, peripheral artery disease*.

* *P < 0.05*.

**Figure 5:**
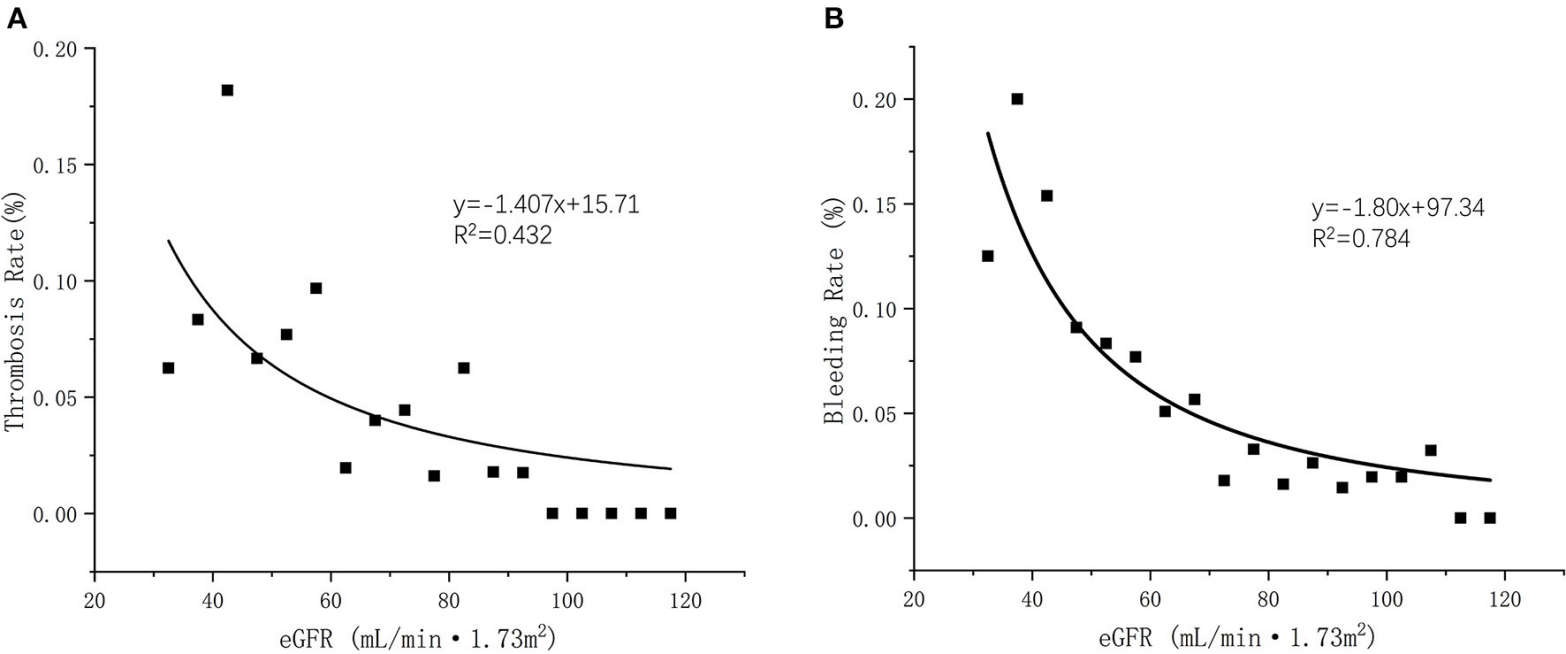
**(A)** Linear regression of eGFR and thrombosis occurrent rate (*R*^2^ = 0.432); **(B)** Linear regression of eGFR and bleeding occurrent rate (*R*^2^ = 0.782).

We applied linear regression to evaluate the relation between eGFR and clinical outcomes. Briefly, either of thrombosis or bleeding rate elevated with the decreased eGFR levels. A positive linear relationship was observed between eGFR levels and occurrence rate of thrombosis and bleeding under anticoagulation patients with dabigatran (*R*^2^ = 0.432 and *R*^2^ = 0.784, respectively) (Figure 5).

## Discussion

In our observational study of 560 patients with NVAF, we found that the utilization of 110 mg dabigatran continued to increase with confirmed clinical effectiveness and safety in this population. The sharp rise in dabigatran prescription was mainly driven by its approval in late 2012 (11). Furthermore, we demonstrated for the first time in the Chinese population that 110 mg dabigatran treatment in patients with moderate renal insufficiency was associated with an increased risk of thromboembolism and bleeding compared with the mild impairment and normal groups. Even though, the absolute incidence of cumulative events gradually increased with the decline of renal function. The individuals enrolled in our study are mostly elderly (Mean age as 67.9 y) and tended to have a high risk of thrombosis. The thrombus events in elderly patients with atrial fibrillation were presented with multi location thrombus. The venous system thrombosis and arterial system thrombosis may occur repeatedly at the same time, and patients have lower extremity deep venous thrombosis, atrial thrombosis and coronary artery thrombosis.

Warfarin has been widely prescribed for thromboembolic stroke prevention in patients diagnosed as NVAF, being considered eligible for anticoagulation for decades (12–14). However, patients with renal dysfunction were at high risk of bleeding attributed to the descending of platelet aggregation and treatment with warfarin could not entrust a thromboembolic risk reduction (15, 16). In the RE-LY and many real world clinical trials, dabigatran showed greater benefit on bleeding risk compared with vitamin K antagonist (VKA) (5). In contrast to VKA, dabigatran is mainly dependent on renal elimination and labeled for the contraindication of severe renal impaired patients with eGFR < 30 mL/min·1.73 m^2^ (17). As a novel oral anticoagulant, dabigatran is given in a fixed dose without frequent monitoring, which makes it more acceptable for patients. However, due to changes in renal function, there were considerable inter-individual variations in pharmacodynamics (PD) of dabigatran-treated patients, which might affect the concentration of dabigatran among different individuals (18). Thus, we need to investigate the influence of renal function on the patient's drug response to dabigatran so as to improve personalized anticoagulation treatment.

This study was the first to investigate the potential safety and effectiveness of 110 mg dabigatran in patients with different levels of kidney function. Our results were based on a real cohort study of patients with non-valvular atrial fibrillation with follow-ups. In this pre-specified analysis concerning the clinical effectiveness of dabigatran in relation to renal function, there were significant interactions primarily for the anti-thrombosis outcomes that were more pronounced when renal function was estimated with the eGFR classification (19, 20). It has been suggested that dabigatran anticoagulant patients with lower eGFR levels tend to have a higher recurrence rate of thrombosis, which may worsen the clinical outcomes. The increasing thrombosis risks with reducing renal function were consistent with previous findings of RELY trial in patients with non-valvular atrial fibrillation, both in terms of the system thrombosis events and all-cause mortality (5, 21–23). Further, a secondary analysis of cumulative thrombosis occurrence estimates demonstrated that lower eGFR subgroups were more likely to experience thrombosis earlier during medication with dabigatran (*P* = 0.021).

Our results showed that gastrointestinal hemorrhage events appeared significant more frequently in the moderately renal impaired group (6.94%) than those in the mildly impaired group (1.54%) and normal group (1.22%) treated with dabigatran. In this study, although no significant difference was found among groups in the incidences of other bleeding complications such as operation site hemorrhage, hematuria, skin ecchymosis, and bleeding gums, individuals in the moderate renal insufficiency group were observably prone to having bleeding events earlier during medication with dabigatran. Our results indicated that impaired kidney function might increase the potential risk of bleeding in patients medicated with dabigatran. Previous studies had acknowledged that decreased kidney function was associated with increased risk of bleeding among patients with atrial fibrillation, due to an elevated risk of drug accumulation (4, 6, 24). One large trial showed that kidney function and age seemed to be highly correlated with plasma dabigatran levels (25). Another study demonstrated that significant increased exposure of dabigatran was detected in patients with moderate and severe renal impairment (26). The mentioned study described that, in patients with moderate kidney impairment, exposure of dabigatran increased 3-fold, while the exposure increased 6-fold in individuals with severely impaired kidney function. Likewise, the mentioned large trial revealed over 2-fold increases in concentrations in moderately renal impaired group compared with patients whose kidney function stratified in mildly impaired or normal level (25). The most common type of bleeding we found was gastrointestinal bleeding, which seemed to be similar to that reported in a previous publication, stating that ~74% of bleeding events associated with dabigatran occurred in the gastrointestinal tract, especially for patients with moderate or mild kidney impairment (7). This finding could be clarified that the tartaric acid coating of dabigatran might pose a straight impact on the intestinal lumen. Dabigatran had also been observed to have some extent of intra-luminal anticoagulant activity due to its incomplete absorption across the gastrointestinal mucosa (27).

Despite favorable anti-thrombosis outcomes with dabigatran as compared with warfarin in many researches (5, 19), there is uncertainty regarding the benefit of composite endpoint in NVAF patients with different stages of chronic kidney disease. Consistent with the retrospective study which suggested the event-free outcomes for patients with lower eGFR levels was significantly shortened (28), our results displayed significant difference regarding composite endpoint among different eGFR groups, whereas we saw a significant trend that patients with impaired kidney function in the dabigatran anticoagulant population might experience shorter event-free outcomes. The moderate renal dysfunction group had a larger proportion of complications due to bleeding and systemic thrombosis, and the difference in anticoagulation-related composite endpoints were significantly evident among the groups.

When interpreting the available data, chronic kidney disease and diabetes were identified as independent predictors of the composite results, contributing prominently to both bleeding and thrombosis events. On one hand, previous evidence demonstrated that renal dysfunction and diabetes increase the likelihood of major adverse cardiovascular events (MACE), and therefore, patients with concomitant CKD and DM could encounter significantly worse outcomes than others (29, 30). On the other hand, many clinical evidences concluded that the increased risk of bleeding in the NOAC anticoagulation population was related to CKD and other comorbidities (23, 31). Data from one study indicated that medication with NOACs in end stage renal disease (ESRD) patients would attribute to higher risk of re-hospitalization or death from composite events when compared with warfarin (32). Moreover, the HAS-BLED score which is currently recommended by European Society of Cardiology (ESC) AF Guidelines for hemorrhagic risk stratification indicated that renal dysfunction is a powerful predictor for further bleeding risk and probably could induce more unwanted events (33).

We acknowledged several limitations in our work. First, owing to the nature of this observational study, we had some relevant limitations such as selection bias or the risk of under-reporting which might have an impact on research quality. Second, we evaluated the clinical outcomes of dabigatran in different renal function status in a pooled analysis of thrombosis and bleeding-associated conditions, which had different acute or chronic thromboembolic risks and usually affected patients with different characteristics. Third, we conducted a limited follow-up for 12 months in small sample size, with a total of 19 thrombotic events which led to a low statistical power for testing interaction. A long-term study was further needed to investigate the impact of renal function on clinical outcomes. Fourth, we performed this study only with 110 mg dabigatran, which might be a limitation. Meanwhile, no drug interactions with dabigatran were found during the follow-up period. Finally, we did not assess the plasma concentration of dabigatran to perform pharmacokinetic modeling and simulation for a recommended dose in patients with severe renal dysfunction, and therefore missed the pharmacokinetic data that could directly predict clinical outcomes.

## Conclusions

Our findings indicated that rates of thrombosis and bleeding increased as renal function deteriorates for AF patients taking low dose dabigatran. The impaired renal function was associated with higher systemic embolism risks over 12-month follow-ups, irrespective of associated comorbidities. As for the safety outcomes, a significantly greater relative increase in bleeding risk was displayed in patients with low eGFR levels.

## Data Availability Statement

The original contributions presented in the study are included in the article/Supplementary Material, further inquiries can be directed to the corresponding author/s.

## Ethics Statement

The studies involving human participants were reviewed and approved by the Ethics Committee of Zhongshan Hospital, Fudan University. The patients/participants provided their written informed consent to participate in this study. Written informed consent was obtained from the individual (s) for the publication of any potentially identifiable images or data included in this article.

## Author Contributions

XL, QL, and QJ contributed to the conception and design of the study. CZ, ZW, and QJ contributed to data acquisition and collection. XL and CZ contributed to the statistical analyses. XL wrote the first draft of the manuscript. All authors contributed to manuscript revision, read, and approved the submitted version.

## Funding

This study was supported by the Shanghai key clinical specialist construction projects (shslczdzk06504) and Shanghai Rising Stars of Medical Talent Youth Development Program–Youth Medical Talents–Clinical Pharmacist Program [SHWJRS (2019)_072].

## Conflict of Interest

The authors declare that the research was conducted in the absence of any commercial or financial relationships that could be construed as a potential conflict of interest.

## Publisher's Note

All claims expressed in this article are solely those of the authors and do not necessarily represent those of their affiliated organizations, or those of the publisher, the editors and the reviewers. Any product that may be evaluated in this article, or claim that may be made by its manufacturer, is not guaranteed or endorsed by the publisher.
